# Differential attrition in randomized controlled trials of digital mental health interventions in the workplace: A systematic review and meta-analysis (EMPOWER H2020 project)

**DOI:** 10.1192/j.eurpsy.2024.338

**Published:** 2024-08-27

**Authors:** C. de Miquel, J. M. Haro, C. M. van der Feltz-Cornelis, A. Ortiz-Tallo, T. Chen, M. Sinokki, P. Naumanen, B. Olaya, R. A Lima

**Affiliations:** ^1^Research, Innovation and Teaching Unit, Parc Sanitari Sant Joan de Déu, Sant Boi de Llobregat; ^2^Centro de Investigación Biomédica en Red de Salud Mental (CIBERSAM), Madrid; ^3^Department of Medicine, University of Barcelona, Barcelona, Spain; ^4^Dept. of Health Sciences, Hull York Medical School, University of York, York, United Kingdom; ^5^Department of Psychiatry, School of Medicine, Universidad Autónoma de Madrid, Madrid, Spain; ^6^Canberra Business School, University of Canberra; ^7^Research School of Population Health, Australian National University, Canberra, Australia; ^8^ Länsirannikon Työterveys Oy; ^9^Department of Occupational Health, University of Turku, Turku, Finland

## Abstract

**Introduction:**

Digital interventions have been found to be successful in preventing occupational mental health concerns, however, they seem to be affected by attrition bias through high attrition rates and differential attrition. Differential attrition arises when the rates of participant dropouts differ across different treatment conditions and is considered a significant challenge to internal validity.

**Objectives:**

We aimed at systematically review and meta-analyse differential attrition of digital mental health interventions in the workplace setting.

**Methods:**

On January 2, 2022, we performed a search in the following electronic databases: PubMed, Scopus, and Web of Science Core. We utilized a combination of terms from five distinct areas, namely mental health, intervention, workplace, implementation, and study design. The study encompassed adult employees who took part in a randomized control trial aimed at preventing mental health issues in the workplace through an online intervention. A team of six reviewers collaborated on the study selection process, while two independent researchers conducted the data extraction for the selected studies. We performed a meta-analysis of the log-transformed relative attrition rates of the included studies using a random-effects model with limited maximum-likelihood (REML) estimation to account for the degree of heterogeneity.

**Results:**

A total of 19 studies were included in the meta-analysis. For baseline to post-intervention, the average total attrition was 26.27% (SD = 21.16%, range = 0 – 66.3%) and the random effects model revealed a higher attrition rate in the intervention group compared to the control group, with a pooled risk ratio of 1.05 (95% CI: 1.01 - 1.10, p = .014). For baseline to follow-up measurement the average total attrition was 27.71% (SD = 20.80%, range = 0 – 67.78%), however, in this case the random effects model did not indicate a higher attrition in the intervention group when compared to the control group (pooled risk ratio = 1.05, 95% CI: 0.98 – 1.12, p = .183).

**Conclusions:**

There is an indication of higher attrition in the intervention group as compared to the control group in occupational e-mental health interventions from baseline to post-intervention, however this does not seem to be the case for baseline to follow-up attrition. These results should be taken into account in the design process of studies and statistical analyses should be adapted to counteract the bias that could result from differential attrition.

**Disclosure of Interest:**

None Declared

